# Prolastin, a pharmaceutical preparation of purified human α1-antitrypsin, blocks endotoxin-mediated cytokine release

**DOI:** 10.1186/1465-9921-6-12

**Published:** 2005-01-31

**Authors:** Izabela Nita, Camilla Hollander, Ulla Westin, Sabina-Marija Janciauskiene

**Affiliations:** 1 Department of Medicine, Lund University, University Hospital Malmö, 20502 Malmö, Sweden; 2 Department of Otolaryngology and Head and Neck Surgery, Lund University, University Hospital Malmö, 20502 Malmö, Sweden

**Keywords:** α1- antitrypsin, Prolastin, monocytes, neutrophils, inflammation, endotoxin

## Abstract

**Background:**

α1-antitrypsin (AAT) serves primarily as an inhibitor of the elastin degrading proteases, neutrophil elastase and proteinase 3. There is ample clinical evidence that inherited severe AAT deficiency predisposes to chronic obstructive pulmonary disease. Augmentation therapy for AAT deficiency has been available for many years, but to date no sufficient data exist to demonstrate its efficacy. There is increasing evidence that AAT is able to exert effects other than protease inhibition. We investigated whether Prolastin, a preparation of purified pooled human AAT used for augmentation therapy, exhibits anti-bacterial effects.

**Methods:**

Human monocytes and neutrophils were isolated from buffy coats or whole peripheral blood by the Ficoll-Hypaque procedure. Cells were stimulated with lipopolysaccharide (LPS) or zymosan, either alone or in combination with Prolastin, native AAT or polymerised AAT for 18 h, and analysed to determine the release of TNFα, IL-1β and IL-8. At 2-week intervals, seven subjects were submitted to a nasal challenge with sterile saline, LPS (25 μg) and LPS-Prolastin combination. The concentration of IL-8 was analysed in nasal lavages performed before, and 2, 6 and 24 h after the challenge.

**Results:**

*In vitro*, Prolastin showed a concentration-dependent (0.5 to 16 mg/ml) inhibition of endotoxin-stimulated TNFα and IL-1β release from monocytes and IL-8 release from neutrophils. At 8 and 16 mg/ml the inhibitory effects of Prolastin appeared to be maximal for neutrophil IL-8 release (5.3-fold, p < 0.001 compared to zymosan treated cells) and monocyte TNFα and IL-1β release (10.7- and 7.3-fold, p < 0.001, respectively, compared to LPS treated cells). Furthermore, Prolastin (2.5 mg per nostril) significantly inhibited nasal IL-8 release in response to pure LPS challenge.

**Conclusion:**

Our data demonstrate for the first time that Prolastin inhibits bacterial endotoxin-induced pro-inflammatory responses *in vitro *and *in vivo*, and provide scientific bases to explore new Prolastin-based therapies for individuals with inherited AAT deficiency, but also for other clinical conditions.

## Background

α1-antitrypsin (AAT) is a glycoprotein, which is the major inhibitor of neutrophil elastase and proteinase 3 [1,2]. AAT is mainly produced in liver cells, but also in extrahepatic cells, such as monocytes, macrophages and pulmonary alveolar cells [3,4]. The average concentration of AAT in plasma in healthy individuals is 1.3 mg/ml, with a half-life of 3 to 5 days. AAT is an acute phase protein, and its circulating levels increase rapidly to concentrations exceeding 2 mg/ml in response to inflammation or infection [5]. Individuals with plasma AAT values below 0.7 mg/ml are considered to be AAT deficient [6,7]. Over 75 alleles of AAT have been identified to date, of which at least 20 affect either the amount or the function of the AAT molecule in vivo [6-8]. A very common deficiency allele is termed Z, which differs from the normal M in the substitution of Glu 342 to Lys [7,9,10]. This single amino acid exchange causes spontaneous polymerization of the AAT, markedly impeding its release into the circulation [11]. The retained material is associated with hepatic diseases [12], while diminished circulating levels lead to antiproteinase deficiency and higher susceptibility to elastase mediated tissue injury [13,14]. The alleles of AAT are inherited in an autosomal codominant manner [2]. Therefore, individuals heterozygous for the Z allele (MZ) have 30–40% whereas individuals homozygous for the Z allele (ZZ) have only 10–15% of normal plasma AAT levels [15-17]. Tobacco smoke and air pollution have long been recognised as risk factors for the development of chronic obstructive pulmonary disease (COPD); the only proven genetic risk factor, however, is the severe Z deficiency of AAT [18,19]. Cigarette smokers with AAT-deficiency develop COPD much earlier in life than smokers with the normal AAT genotype [8,10,11].

The pulmonary emphysema that is associated with inherited AAT deficiency is intimately linked with the lack of proteinase inhibitor within the lungs that is available to bind to, and inactivate, neutrophil elastase. On the basis of clinical observations involving patients with inherited AAT deficiency and various experimental studies, the elastase-AAT imbalance hypothesis became widely accepted as the explanation for lung tissue destruction in emphysema [20,21]. There is now increasing evidence that an excessive activity of various proteolytic enzymes in the lung milieu, including members of the serine, cysteine and metalloprotease families, may damage the elastin network of lungs [14]. Since the severe ZZ and intermediate MZ AAT deficiency accounts for less than 1–2% and 8–18% of emphysema cases, it is believed that the protease-antiprotease hypothesis provides a rational basis for the explanation of the development and progression of emphysema in general [22,23].

Based on the protease-antiprotease hypothesis, augmentation therapy of emphysema with severe AAT deficiency was introduced during the 1980s [24]. Intravenous administration of a pasteurized pooled human plasma AAT product (Prolastin; Bayer Corporation; Clayton, North Carolina) is used to increase AAT levels in deficient individuals [25]. The major concept behind augmentation therapy is that a rise in the levels of blood and tissue AAT will protect lungs from continuous destruction by proteases, particularly neutrophil elastase [26]. For example, anti-elastase capacity in the lung epithelial lining fluid has been found to increase to 60–70% of normal in homozygous Z AAT-deficient individuals subjected to augmentation therapy [26,27]. Whether this biochemical normalization of AAT levels influences the pathogenic processes of lung disease is still under debate. The most recent results, however, suggest that Prolastin therapy may have beneficial effects in reducing the frequency of lung infections and reducing the rate of decline of lung function [28,29].

There is growing evidence that AAT, in addition to its anti-proteinase activity, may have other functional activities. For example, AAT has been demonstrated to stimulate fibroblast proliferation and procollagen synthesis [30], to up-regulate human B cell differentiation into IgE-and IgG4-secreting cells [31], to interact with the proteolytic cascade of enzymes involved in apoptosis [32,33] and to express contrasting effects on the post-transcriptional regulation of iron between erythroid and monocytic cells [34]. AAT is also known to inhibit neutrophil superoxide production [35], induce macrophage-derived interleukin-1 receptor antagonist release [36] and reduce bacterial endotoxin and TNFα-induced lethality *in vivo *[37,38]. We recently demonstrated, *in vitro*, that both native (inhibitory) and non-inhibitory (polymerised and oxidised) forms of AAT strongly inhibit lipopolysaccharide-induced human monocyte activation [39]. AAT appears to act not just as an anti-proteinase, but as a molecule with broader anti-inflammatory properties. Data presented in this study provide clear evidence that Prolastin, a preparation used for AAT deficiency augmentation therapy, significantly inhibits bacterial endotoxin-induced pro-inflammatory cell responses *in vitro*, and suppresses nasal IL-8 release in lipopolysaccharide-challenged individuals, *in vivo*.

## Materials and Methods

### α1-antitrypsin (AAT) preparations

α1-antitrypsin (Human) Prolastin^® ^(Lot 26N3PT2) was a gift from Bayer (Bayer Corporation, Clayton, North Carolina, USA). This vial of Prolastin contained 1059 mg of functionally active AAT, as determined by capacity to inhibit porcine pancreatic elastase. Prolastin was dissolved in sterile water for injections provided by manufacture and stored at +4°C. Purified human AAT was obtained from the Department of Clinical Chemistry, Malmö University Hospital, Sweden. Native AAT was diluted in phosphate buffered saline (PBS), pH 7.4. To ensure the removal of endotoxins, AAT was subjected to Detoxi-Gel AffinityPak columns according to instructions from the manufacturer (Pierce, IL, USA). Purified batches of AAT were then tested for endotoxin contamination with the Limulus amebocyte lysate endochrome kit (Charles River Endosafe, SC, USA). Endotoxin levels were less than 0.2 enzyme units/mg protein in all preparations used. The concentrations of AAT in the endotoxin-purified batches were determined according to the Lowry method [40]. Polymeric AAT was produced by incubation at 60°C for 10 h. Polymers were confirmed on non-denaturing 7.5% PAGE gels.

### Monocyte isolation and culture

Monocytes were isolated from buffy coats using Ficoll-Paque PLUS (Pharmacia, Sweden). Briefly, buffy coats were diluted 1:2 in PBS with addition of 10 mM EDTA and layered on Ficoll. After centrifugation at 400 *g *for 35 min, at room temperature, the cells in the interface were collected and washed 3 times in PBS-EDTA. The cell purity and amount were determined in a cell counter Autocounter AC900EO (Swelabs Instruments AB, Sweden). The granulocyte fractions were less than 10%. Cells were seeded into 12-well cell culture plates (Nunc, Denmark) at a concentration of 4 × 10^6 ^cells/ml in RPMI 1640 medium supplemented with penicillin 100 U/ml; streptomycin 100 μg/ml; non-essential amino acids 1×; sodium pyruvate 2 mM and HEPES 20 mM (Gibco, UK). After 1 h 15 min, non-adherent cells were removed by washing 3 times with PBS supplemented with calcium and magnesium. Fresh medium was added and cells were stimulated with lipopolysaccharide (LPS, 10 ng/ml, J5 Rc mutant; Sigma, Sweden) in the presence or absence of various concentrations of Prolastin (0–16 mg/ml), constant concentration of native or polymerised AAT (0.5 mg/ml) for 18 h at 37°C, 5% CO_2_.

### Neutrophil isolation and culture

Human neutrophils were isolated from the peripheral blood of healthy volunteers using Polymorphprep TM (Axis-Shield PoC AS, Oslo, Norway) as recommended by the manufacture. In brief, 25 ml of anti-coagulated blood was gently layered over the 12.5 ml of Polymorphprep TM and centrifuged at 1600 rpm for 35 min. Neutrophils were harvested as a low band of the sample/medium interface, washed with PBS, and residual erythrocytes were subjected to hypotonic lysis. Purified neutrophils were washed in RPMI-1640- Glutamax-1 medium (Gibco-BRL Life Technologies, Grand Island, NY) supplemented with 0.1% bovine serum albumin (BSA) and resuspended in the same medium. The neutrophil purity was more than 75% as determined on an AutoCounter AC900EO. Cell viability was > 95% according to trypan blue staining.

Neutrophils (5 × 10^6 ^cells/ml) were plated into sterile ependorf tubes. Zymosan was boiled, washed and sonicated. Opsonized zymosan was prepared by incubating zymosan with serum (1:3) in 37°C water bath for 20 min. After, zymosan was centrifuged, washed with PBS and re-suspended at 30 mg/ml. Cells alone or activated with zymosan (0.3 mg/ml) were exposed to various concentrations of Prolastin (0–8 mg/ml), and native or polymerised AAT preparations (0.5 mg/ml) for 18 h at 37°C 5% CO_2_. Cell free supernatants were obtained by centrifugation at 300 *g *for 10 min, and stored at -80°C until analysis

### Cytokine/chemokine analysis

Cell culture supernatants from monocytes and neutrophils stimulated with LPS or zymosan alone or in combination with Prolastin, native or polymerised AAT were analysed to determine TNFα, IL-1β and IL-8 levels by using DuoSet ELISA sets (R&D Systems, MN, USA; detection levels 15.6, 3.9, and 31.2 pg/ml, respectively).

### Subjects

Seven subjects (four females and three males) of 26–50 (median 38) years of age, non-smokers, non-allergic volunteers participated in the study. All subjects gave written informed consent before participation in the study. None of the subjects has a history of respiratory disease and none took any medication at the study time.

### Study Design

At 2-week intervals each subject was submitted to a nasal challenge with sterile saline, LPS and LPS-Prolastin combination. All experimental sessions were done in the same room. On each provocation day, the nose was inspected and cleaned with 8 ml of isotonic NaCl. Between nasal lavages the subjects stayed in the same building and asked to keep away from known sources of nasal irritants. The night was spent in their own homes. All participants completed a symptom questionnaire. In the first session, the baseline lavage was taken after instillation to each nostril of 8 ml of sterile isotonic NaCl. In the next session, the subjects were challenged with LPS from Escherichia coli serotype 026:B6, Lot 17H4042 (Sigma-Aldrich, USA). The provocation solution was prepared prior to use. LPS was added to 8 ml of sterile 0.9% NaCl to obtain a final concentration of 250 μg/ml, and 100 μl of the provocation solution was sprayed into each nostril, using a needle-less syringe. In the third session, the subjects were first challenged with LPS, as described above, and after 30 min with 2.5 mg of Prolastin into each nostril. Lavage samples were taken with instillation to each nostril of 8 ml of sterile isotonic NaCl after 2, 6 and 24 h followed by assessment of symptoms by a questionnaire. All subject completed a symptom questionnaire with questions about nasal and eye irritation, and throat and airway symptoms. None of the participants reported symptoms of nasal, eye or throat irritations, and no general symptoms such as muscle pain, shivering, were mentioned.

### Nasal Lavage

The procedure for nasal lavage was performed according to a method described by Wihl and co-workers [41]. Each nasal cavity was lavaged separately with a syringe (60 ml) to which a plastic nasal olive was connected for close nostril fitting. To prevent lavage spilling into the throat, the subject was bent forward at an angle of 60° during the procedure. Equilibrium was maintained between the mucosal lining and the lavage fluid by injecting the saline gently into the nasal cavity and drawing it back five times into the syringe. The lavage was performed in both nostrils and samples were collected into a test tube. The samples were then centrifuged at 1750 rpm, 6°C for 10 min and immediately frozen at -80°C. The protein concentration in the lavage fluids was measured by Lowry method and IL-8 levels were determined by DuoSet ELISA sets (R&D Systems, MN, USA; detection levels 31.2 pg/ml).

### Statistical Analysis

Statistical Package (SPSS for Windows, release 11.5, SPSS Inc., Chicago) was used for the statistical calculations. The differences in the means of cell culture experimental results were analysed for their statistical significance with the one-way ANOVA combined with a multiple-comparisons procedure (Scheffe multiple range test). The equality of means of experimental results in healthy volunteers were analysed for statistical significance with independent two sample t-test and repeated measures of ANOVA using the SPSS MANOVA procedure . Tests showing p < 0.05 were considered to be significant.

## Results

### Concentration-dependent effects of Prolastin on LPS-induced cytokine release from human monocytes

**Figure 1:**
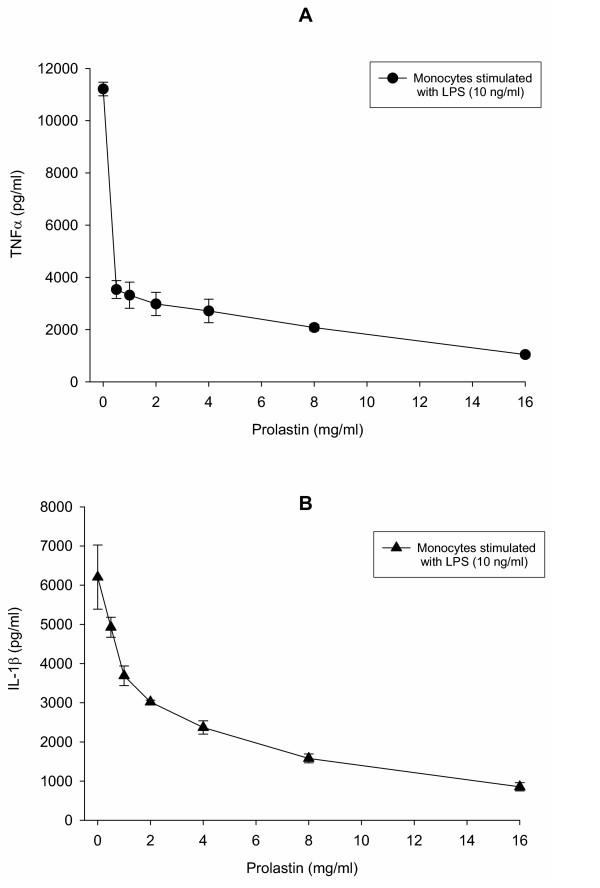
A concentration-response inhibition of lipopolysaccharide-stimulated TNFα (A) and IL-1β (B) release by Prolastin in human blood monocytes. Isolated blood monocytes were treated with LPS (10 ng/ml) alone or together with various concentrations of Prolastin (0–16 mg/ml) for 18 h. TNFα and IL-1β levels were measured by ELISA. Data are the means of quadruplicate culture supernatants ± S.E. and are representative of three separate experiments.

Various concentrations of Prolastin (0–16 mg/ml) were added to adherent-isolated human monocytes with or without LPS (10 ng/ml). Cells stimulated with LPS alone served as a positive control, while PBS stimulated monocytes served as negative controls. As illustrated in figures 1A and 1B, simultaneous incubation of monocytes with LPS and Prolastin resulted in a reduction in TNFα and IL-1β release compared to the cells stimulated with LPS alone. Inhibition of LPS-induced cytokine release by Prolastin was concentration-dependent and was typically observed over a concentration range of 0.5–16 mg/ml. At 16 mg/ml the inhibitory effects of Prolastin appeared to be maximal for both TNFα (10.7-fold, p < 0.001) and IL-1β (7.3-fold, p < 0.001), compared to LPS alone.

### Inhibitory effects at 0.5 mg/ml of AATs on LPS-mediated IL-1β and TNFα release

**Figure 2:**
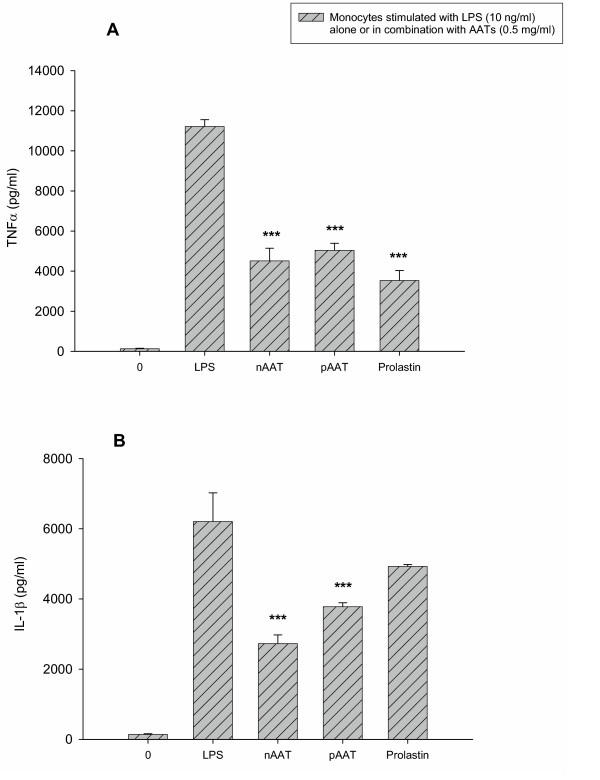
Comparisons of the effects of native (nAAT), polymeric (pAAT) and Prolastin on lipopolysaccharide – stimulated TNFα (A) and IL-β (B) production by human blood monocytes isolated from four healthy donors. Isolated blood monocytes were treated with LPS (10 ng/ml) alone or together with 0.5 mg/ml nAAT, pAAT or Prolastin for 18 h. TNFα and IL-1β levels were measured by ELISA. Each bar represent the mean ± S.E. *** p < 0.001.

We recently found that simultaneous incubation of monocytes with LPS and either the inhibitory (native) or non inhibitory (polymeric) form of AAT resulted in a reduction in TNFα and IL-1β release compared to the cells stimulated with LPS alone. At 0.5 mg/ml the effects of native and polymerised AAT appeared to be maximal (41). Therefore, we selected a 0.5 mg/ml concentration of Prolastin, native and polymerised AAT, and compared their effects on LPS-stimulated cytokine release at 18 h. As shown in figures 2A and 2B, LPS triggered a significant release of TNFα and IL-1β (p < 0.001 v medium alone) by monocytes. At 0.5 mg/ml, native and polymerised AAT remarkably inhibited LPS-induced TNFα and IL-1β release (p < 0.001) (Fig. 2). The inhibitory effect of Prolastin (0.5 mg/ml) on LPS-stimulated TNFα release was comparable in magnitude to that of native or polymeric AAT, whereas its inhibitory effect on LPS-induced IL-1β release did not reach significance.

### Concentration-dependent effects of Prolastin on neutrophil IL-8 release

**Figure 3:**
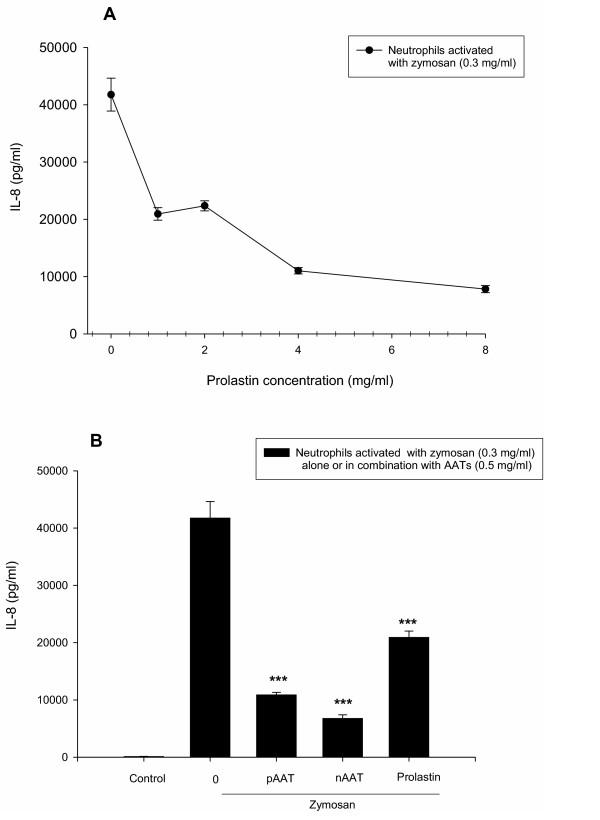
Effects of AATs on neutrophils activated with zymosan. (A) Concentration-dependent effects of Prolastin on IL-8 release from neutrophils activated with opsonised zymosan. Freshly isolated blood neutrophils were treated with zymosan (0.3 mg/ml) alone or together with various concentrations of Prolastin (0–8 mg/ml) for 18 h. IL-8 levels were measured by ELISA. Data are the means of quadruplicate culture supernatants ± S.E. and are representative of three separate experiments. (B) Effects of opsonised zymosan alone or together with native (nAAT), polymeric (pAAT) AAT or Prolastin on IL-8 release from neutrophils. The release of neutrophil IL-8 was measured in cell free supernatants as described in Materials and methods. Neutrophils were treated for 18 h with a constant amount of zymosan (0.3 mg/ml) alone or together with nAAT, pAAT or Prolastin (0.5 mg/ml) for 18 h. IL-8 levels were measured by ELISA. Each bar represents the means ± S.E. of three separate experiments carried out in duplicate repeats. *** p < 0.001

The effects of Prolastin (0–8 mg/ml) on human neutrophil IL-8 production are shown in Figure 3A. Neutrophils stimulated with opsonized zymosan (0.3 mg/ml) released a large amount of IL-8 (p < 0.001), compared to controls. Prolastin inhibited IL-8 release by neutrophils stimulated with opsonized zymosan (Fig 3A). This inhibition was concentration-dependant, with maximal suppression of IL-8 release (5.3-fold, p < 0.001 compared to zymosan treated cells) at 8 mg/ml.

### Inhibitory effects at 0.5 mg/ml of native, polymeric AAT and Prolastin on zymosan-mediated IL-8 release

Neutrophils were stimulated with zymosan (0.3 mg/ml) or AATs (0.5 mg/ml) either alone or in combination for 18 h and IL-8 protein determined. As illustrated in figure 3B, polymeric and native AAT and Prolastin significantly inhibited the release of IL-8 protein by activated neutrophils. In terms of maximal effect, native AAT >polymerised AAT>Prolastin. It must be noted that native, polymeric AAT and Prolastin alone showed no effect on neutrophils, relative to non-treated buffer controls (data not shown).

### Inhibition of the LPS-induced increase in nasal IL-8 release by Prolastin

**Figure 4:**
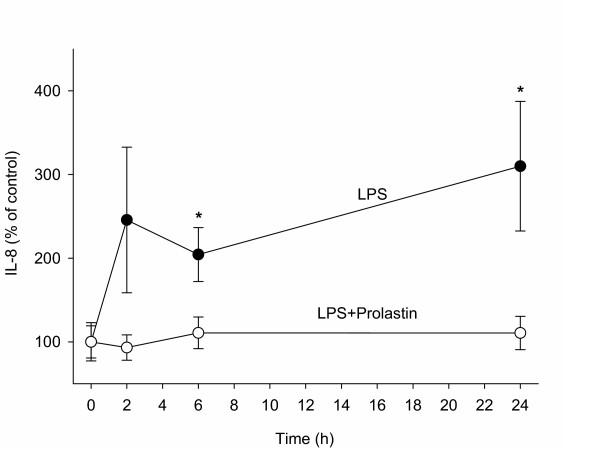
IL-8 analysis in nasal lavage of subjects challenged with LPS alone or LPS+Prolastin combination. Seven healthy volunteers were treated with LPS (25 μg/nostril) or with LPS followed 30 min later with Prolastin (2.5 mg/nostril), nasal lavage was collected at different time points (0, 2, 6 and 24 h) as described in Material and Methods. The concentration of IL-8 (pg/ml) was measured by ELISA. IL-8 values are expressed as a ratio of IL-8 concentration at selected time point and the basal level. Independent two sample t-test shows after 6 and 24 h significantly higher levels of IL-8 in subjects treated with LPS compared to LPS+Prolastin. * p < 0.05

To assess the effect of Prolastin on LPS-induced nasal provocation, IL-8 levels in nasal lavages were measured. Nasal instillation 25 μg per nostril of LPS alone or in combination with 2.5 mg/ml of Prolastin was performed in non-smoking and non-allergic volunteers (n = 7, 4 females and 3 males). The IL-8 release in response to LPS challenge increased over time compared to baseline levels (Fig. 4). The levels of IL-8 increased already after 2 h of LPS challenge (245.7% ± 87) and remained higher after 24 h (310 ± 77.5) compared to baseline (100% ± 19.2). By contrast, when IL-8 levels were examined in LPS-Prolastin-treated lavage samples, no significant changes in IL-8 release were observed compared to baseline. In the presence of Prolastin, the LPS effect on IL-8 release was inhibited (p < 0.05) (Fig. 4).

## Disscussion

There is now, however, ample evidence that serine proteinase inhibitors (serpins), in addition to their well established anti-inflammatory capacity to regulate serine proteinases activity, may possess broader anti-inflammatory properties. Several studies have shown that the biological responses of bacterial lipopolysaccharide (endotoxin) *in vivo *may be sensitive to serpins. For example, the serpin antithrombin, has been shown to protect animals from LPS-induced septic shock and also to inhibit IL-6 induction by LPS [42,43]. Our recent study provided first *in vitro *evidence that native (inhibitor) and at least two modified (non-inhibitory i.e. polymeric and oxidised) forms of AAT can block the release of an array of chemokine and cytokines from LPS-stimulated monocytes [39]. These studies therefore further support a central role of serpins in inflammation, not only as the regulators of proteinase activity, but also as the suppressers of endotoxin induced pro-inflammatory responses. In line with these findings, we demonstrate here that Prolastin, a preparation of human AAT which is used for augmentation therapy, significantly inhibits endotoxin-induced pro-inflammatory effects *in vitro *and *in vivo*.

Stimulation of human monocytes and neutrophils with bacterial endotoxin results in the release of a range of inflammatory mediators including the pro-inflammatory cytokines (*e.g. *IL-6, IL-1β and TNFα) and the chemokines (*e.g. *MCP-1 and IL-8) [44-46]. Together, these play a crucial role in the recruitment and activation of leukocytes and the subsequent release of harmful proteases that may further perpetuate the inflammatory process. We found that Prolastin significantly inhibits endotoxin-induced IL-1β and TNFα release by monocytes and IL-8 release by neutrophils *in vitro*. The Prolastin exhibited these anti-inflammatory properties in a concentration-dependent manner. Its maximal effects were observed with 16 mg/ml in the monocyte model and with 8 mg/ml in the neutrophil model, since doubling these concentrations did not significantly modify the intensity of the effects. Indeed, Prolastin markedly prevented endotoxin-induced cell activation at 0.5–4 mg/ml concentrations, implying that these lower concentrations of Prolastin might also be sufficient to inhibit endotoxin effects. It is worth noting that in order to reduce a potential risk of transmission of infectious agents the Prolastin preparation is heat-treated in solution at 60° ± 0.5 for not less than 10 h. Data from *in vitro *studies show that heat-treatment results in AAT polymerization and loss of its inhibitory activity [47,48]. Therefore, in our experimental model we compared anti-inflammatory effects of Prolastin with those of native and heat treated (60°C 10 h) AATs. At concentrations used (0.5 mg/ml), no significant difference was found between the effects of Prolastin and native or heat-treated (polymeric) AAT on endotoxin-induced monocyte TNFα and neutrophil IL-8 elevation. The median concentrations of endotoxin-stimulated IL-1β levels also decreased in the presence of Prolastin but failed to reach statistical significance. In general, inhibitory effects on endotoxin-stimulated monocyte IL-1β and neutrophil IL-8 release were better pronounced by native AAT compared to polymeric AAT or Prolastin. Similarly, in our previous study we found that in terms of maximal effect, native AAT >polymerised AAT>oxidized AAT were efficient in inhibiting LPS-stimulated TNFα and IL-1β, and IL-8 release from monocytes [39]. Further studies will be necessary to better evaluate how temperature, pH or other physicochemical challenges may influence anti-inflammatory effectiveness of AAT preparations.

To explore our hypothesis that AAT functions as a potent inhibitor of endotoxin-induced effects, we examined whether Prolastin also inhibits responses to LPS in the nasal airway, *in vivo*. In particular, we were interested in concentrations of the neutrophil chemoattractant, IL-8. Endotoxin (or LPS) from gram-negative bacteria is a common air contaminant in a number of occupational conditions, especially those in which exposure to animal waste or plant matter occurs [44,49-51]. Levels of LPS in such environments may exceed 20 μg/m^3 ^air and may be associated with respiratory symptoms and nasal inflammation in exposed persons [52]. For example, nasal inflammation as evaluated by an increased influx of inflammatory cells into the nasal airway and increased IL-8 levels, has been described in persons occupationally exposed to LPS [51]. Moreover, it has been suggested that constitutive levels of IL-8 might further enhance responses to an inflammatory stimulus, such as LPS [53]. A number of experimental studies have shown that a nasal instillation of LPS causes the cytokine and chemokine reaction [54,55]. In our pilot study we also showed that instilled defined amounts of endotoxin (25 μg/per nostril) induce time-dependent nasal IL-8 release in normal subjects. Two hours after LPS instillation the IL-8 levels in nasal lavage reached more than twice the basal level and remained higher during all the times studied. However, during the next session, when 30 min after challenge with LPS, Prolastin (2.5 mg/ per nostril) was instilled, no induction of nasal IL-8 release was found compared to the basal levels. Furthermore, the protective ability of Prolastin did not disappeared over study time. We cannot determine from these experiments whether Prolastin is directly suppressing IL-8 release or suppressing another inflammatory response that leads to IL-8 release; nonetheless, our finding suggests that effects of Prolastin directed against endotoxin-stimulated inflammatory responses may be beneficial.

Thus, data from both *in vitro *and *in vivo *experiments provide novel evidence that the Prolastin preparation is a potent inhibitor of endotoxin effects. The major concept behind augmentation therapy with pooled plasma-derived AAT has been that a rise in the level of AAT in subjects with severe inherited AAT deficiency would protect the lung tissue from continued destruction by proteinases (i.e. primarily leukocyte elastase) [7,56,57]. Recent findings provide evidence that augmentation therapy with AAT reduces the incidence of lung infections in patients with AAT-related emphysema [28,58]. Furthermore, Cantin and Woods have reported that aerosolized AAT suppresses bacterial proliferation in a rat model of chronic *Pseudomonas aeruginosa *lung infection [59]. Stockley and co-workers demonstrated that a short-term therapy of AAT augmentation not only restores airway concentrations of AAT to normal, but also reduces levels of leukotriene B4, a major mediator of neutrophil recruitment and activation. Interestingly, authors have suggested that the efficacy of AAT augmentation may be most beneficial in individuals with the most inflammation [29,60]. Data presented in this study clearly show that Prolastin inhibits endotoxin-stimulated pro-inflammatory responses, and thus provides new biochemical evidence supporting the efficacy of augmentation therapy. The current findings also suggest that Prolastin may, in fact, be used for broader clinical applications than merely augmentation therapy.

## Abbreviations

AAT, α1-antitrypsin; COPD, chronic obstructive pulmonary disease; LPS, lipopolysaccharide; ZZ, homozygous AAT-deficiency variant; MM, wild type AAT variant; PBS, phosphate buffered saline; EDTA, ethylenediaminetetraacetic acid; HEPES, 4-(2-hydroxyethyl)-1-piperazineethanesulfonic acid

## Authors' contribution

Izabela Nita, performed cell culture experiments, made contribution to acquisition of data;

Camilla Hollander, made substantial contribution to patient study design, material collection and analysis; Ulla Westin, contributed to study design and data interpretation; Sabina Janciauskiene, contributed to conception and study design, data interpretation and wrote the article
